# Clinical, Pathological, and Molecular Characteristics Correlating to the Occurrence of Radioiodine Refractory Differentiated Thyroid Carcinoma: A Systematic Review and Meta-Analysis

**DOI:** 10.3389/fonc.2020.549882

**Published:** 2020-09-30

**Authors:** Yi Luo, Hongyi Jiang, Weibo Xu, Xiao Wang, Ben Ma, Tian Liao, Yu Wang

**Affiliations:** ^1^Department of Head and Neck Surgery, Fudan University Shanghai Cancer Center, Fudan University, Shanghai, China; ^2^Department of Oncology, Shanghai Medical College, Fudan University, Shanghai, China

**Keywords:** thyroid cancer, radioactive iodine refractory (RAIR), poorly differentiated thyroid cancer, risk factors, meta-analysis

## Abstract

**Background:** Recently, radioiodine refractory differentiated thyroid cancer (RR-DTC) has received increasing attention due to its poor prognosis. The roles of clinical, pathological, and molecular features in the development of RR-DTC remain controversial and require additional investigation. This study aimed to evaluate the association between these risk factors and the occurrence of RR-DTC.

**Methods:** We performed a systematic search for relevant literature following the recommendations of the Preferred Reporting Items for Systematic Reviews and Meta-Analyses (PRISMA) in PubMed, EMBASE, Medline, SCOPUS, and Web of Science up to the July 15, 2020. Observational studies that investigated the risk factors for RR-DTC were included. Fixed- or random-effects models were used to calculate pooled odds ratios (ORs) or mean differences (MD) with corresponding 95% confidence intervals.

**Results:** We included 13 eligible studies incorporating 1,431 cases, of which 603 were patients with RR-DTC. The pooled analysis indicated that four parameters significantly increased the risk of RR-DTC: extrathyroidal extension (ETE) (OR: 2.28, 95% CI: 1.43–3.64, *I*^2^ = 14%), *BRAF*^*V*600*E*^ mutation (OR: 3.60, 95% CI: 1.74–7.46, *I*^2^ = 69%), *TERT* promoter mutation (OR: 9.84, 95% CI: 3.60–26.89, *I*^2^ = 61%) and high-risk histological subtype (OR: 1.94, 95% CI: 1.15–3.27, *I*^2^ = 15%), including tall cell variant papillary thyroid carcinoma (PTC), sclerosing diffuse PTC, hobnail variant PTC, follicular thyroid carcinoma (FTC) (including Hürthle cell), and poorly differentiated thyroid carcinoma (PDTC). However, there was no statistical significance regarding sex, age, tumor size, multifocality, or lateral lymph node metastasis. Subgroup and sensitivity analyses were conducted to further confirm the robustness of the results.

**Conclusions:** Histological subtype, ETE, *BRAF*^*V*600*E*^ mutation, and *TERT* promoter mutation could be considered clinicopathological factors and biomarkers. They could assist in risk stratification, prognostic prediction, and individual therapy options for RR-DTC.

## Introduction

In recent decades, thyroid cancer (TC) has emerged as a striking health issue, and the global incidence of TC is 6.7 per 100,000 (1). Papillary thyroid carcinoma (PTC) and follicular thyroid carcinoma (FTC) are collectively characterized as differentiated thyroid carcinoma (DTC) and account for more than 90% of all thyroid malignancies. Most DTC cases can be treated successfully by thyroidectomy, selective radioactive iodine (RAI) therapy, and thyroid stimulating hormone (TSH)-suppressive therapy and have a favorable prognosis. However, the incidences of local recurrence and distant metastases are ~30 and 10% (2), respectively. Among these patients, unfortunately, one third show initial or gradual loss of iodine uptake and even a decrease in sodium iodide symporter (NIS) expression in the plasma membrane, indicating a status of dedifferentiation known as RAI-refractory DTC (RR-DTC) (3). A long-term study showed that the 10- and 15-year survival rates of patients without any radioiodine uptake were much lower than those of patients with RAI uptake (10 vs. 56%, and 6 vs. 45%) (4).

The American Thyroid Association (ATA) Management Guidelines for Adult Patients with Thyroid Nodules and Thyroid Cancer has classified RR-DTC in four basic ways: (i) the malignant/metastatic tissue has no concentrated RAI, (ii) the tumor tissue has lost the ability to concentrate RAI after previous evidence of RAI-avid disease, (iii) RAI is concentrated in some lesions but not in others, and (iv) metastatic disease progresses despite significant concentration of RAI (5). There is an increasing demand to understand this kind of cancer better and to predict the response to RAI therapies earlier in the disease course. Clinicopathological characteristics as well as molecular features are meaningful indicators that could be utilized for further characterization and prognostication of this tumor. To provide a reliable reference for the prediction of RR-DTC, we conducted this meta-analysis.

## Materials and Methods

The study was carried out according to the Preferred Reporting Items for Systematic Reviews and Meta-Analyses (PRISMA) criteria.

### Search Strategy

The search for eligible studies was conducted in PubMed, EMBASE, Medline, SCOPUS, and Web of Science up to July 15, 2020, with English restriction. The following terms were used: “thyroid neoplasms” or “thyroid carcinoma” or “thyroid cancer” or “thyroid tumor,” “iodine radioisotopes” or ^131^I or radioiodine or “radioactive iodine,” refractory or negative or resistant^*^ or fail^*^ or resist, and predict^*^ or factor or character^*^ or feature or risk. The reference lists of relevant studies and review articles were hand-searched to identify any potential additional relevant articles. The literature search was conducted independently by two investigators, and any inconsistencies were resolved by consensus with a third investigator.

### Selection Criteria

The inclusion criteria of the literature in this meta-analysis were as follows: (1) the original research; (2) observational studies designed to evaluate the association between clinical, pathological, or molecular factors and the development of RR-DTC, including retrospective and prospective studies; (3) patients with RR-DTC were well-defined, and the data of the control group were available; and (4) the data of variables reported could be used to calculate the log odds ratio (OR) or mean difference (MD) with 95% confidence interval (CI). Studies were excluded in cases of duplicated, unavailable, or incomplete data. Reviews, case reports, letters, editorials, and expert opinions were also excluded.

### Data Extraction and Study Assessment

Two investigators (Y.L. and H.J.) extracted key data from the included articles in a standardized Excel sheet independently, and a third independent investigator (B.M.) checked the extracted data. For each article, data were extracted on the authors, year of publication, country, study type, recruitment period, sample size, demographics (age and sex), histological subtype, case number of patients in the RAI refractory (RAIR) group and the RAI-avid (RAIA) group, and RAIR classifications. Disagreements during data extraction were resolved through discussion among all authors. Study quality was assessed by two investigators (Y.L. and W.X.) using the Newcastle-Ottawa Scale (NOS). A third reviewer (X.W.) was available for mediation. The NOS assigns a maximum of 9 points based on three quality parameters, including selection, comparability, and outcome. A score ≥ 7 indicates good quality.

### Statistics Analysis

Risk factors were included in the meta-analysis when they were reported in more than two studies. We considered only adjusted estimates to minimize the impact of confounding factors on pooled effect measures. Thus, for categorical variables, we entered the ratio measures of the (adjusted) effect as a log OR and the standard error (SE) of the log OR using the generic inverse-variance weighting method (6). The mean and standard deviation (SD) of continuous variables were also included in the study. All log ORs and MDs with 95% CIs were calculated. Generally, a fixed- or random-effects model was chosen depending on the heterogeneity, which was regarded as no significance between 25 and 50%, moderate degree between 50 and 75%, and high degree >75%. Different models can lead to quite different outcomes when the heterogeneity is considerable (*I*^2^ > 50%, according to the *Cochrane Handbook*), but this difference will be very slight with data of insignificant heterogeneity. Thus, we conservatively conducted random models for all data to make the results as reliable as possible. In this meta-analysis, subgroup analyses were conducted to explore the source of obvious heterogeneity, and a sensitivity analysis was performed to evaluate the reliability of the associations. A publication bias test was not conducted due to the small sample size. A *p* < 0.05 was considered statistically significant. All analyses were performed with R software version 3.6.2.

## Results

### Search Results and Characteristics of Patients

**Figure 1:**
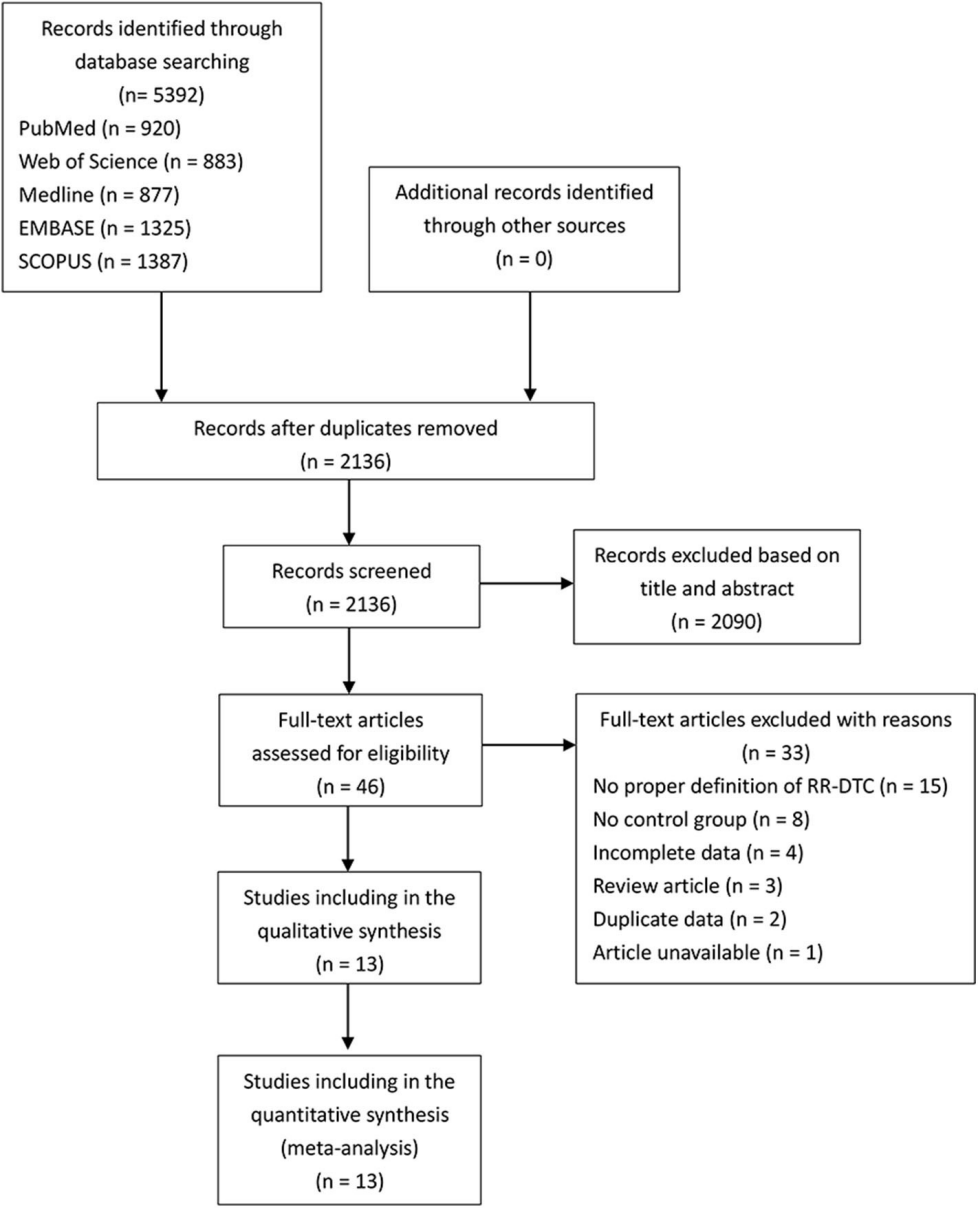
Flow chart of the process of the study selection.

After duplicate studies were excluded, 2,136 articles from PubMed, EMBASE, Medline, SCOPUS, and Web of Science were screened. A total of 2,090 articles were inappropriate for our study according to the title and abstracts. Forty-six remaining studies were retrieved for assessment, and a flow chart showed the process of literature retrieval (Figure 1). Of 13 eligible articles, 12 were retrospective studies, and one study from China was prospective (7–19). Two appropriate reports were included, although they were conference abstracts. Among these 13 studies, five were conducted in China, two in France, two in Korea, two in the United States, and one each in Italy and Germany. There were 603 RR-DTCs and 828 controls involved in the present study. The basic characteristics and NOS results of the identified studies are shown in Table 1 and Supplementary Table 1.

**Table 1:** Characteristics of the selected studies.

**Author/year**	**Country**	**Study type**	**Recruitment period**	**Case number**	**Mean, SD^**1**^ (y)**	**Sex, female/male**	**Histological subtype**	**RR-DTC^**2**^/control**	**RAIR classifications^**3**^**	**NOS^**4**^**
Yang et al. (8)	China	Case control	2008–2016	66	NA	38/28	PTC,^6^ FTC^7^	33/33	a, b, c, d	7
Choi et al. (9)	Korea	Case control	NA^5^	108	41.73, 16.00	NA	PTC	26/82	d, e	6
Gao et al. (10)	China	Prospective cohort	NA	72	NA	56/16	PTC	13/59	c, d, e	8
Meng et al. (11)	China	Case control	2003–2017	67	53.30, 12.22	42/25	PTC	33/34	b, c, e	7
Shobab et al. (12)	USA	Case control	2013–2017	76	57, 15	37/39	PTC, FTC	54/22	a, b, c, d	7
Wang et al. (13)	China	Case control	2008–2015	115	43.04, 14.31	78/37	PTC, FTC	50/65	a, d, e	7
Collina et al. (14)	Italy	Case control	2004–2013	110	NA	84/26	PTC	13/97	a, b, e, f	7
Fouchardiere et al. (16)	France	Case control	2000–2010	63	60.22, 17.84	NA	PDTC^8^	30/33	c, d, e	7
Li et al. (17)	China	Case control	2012–2016	336	41.23, 12.16	244/92	PTC, FTC	112/224	a, b, c, d	7
Wassermann et al. (18)	France	Case control	1990–2011	153	NA	95/58	PTC, FTC, PDTC	91/62	c, d, e	7
Binse et al. (7)	Germany	Case control	NA	40	NA	NA	PTC	20/20	c, f	7
Liu et al. (15)	USA	Case control	1990–2015	164	50 (35–62)^9^	104/60	PTC	103/61	b, c	7
Jung et al. (19)	Korea	Case control	2006–2018	61	NA	42/19	PTC, FTC, PDTC	25/36	c, d, e	7

1 *SD: standard deviation*.

2 *RR-DTC: radioactive iodine refractory differentiated thyroid cancer*.

3 *Radioactive iodine refractory (RAIR) classifications: (a) The malignant/metastatic tissue has never concentrated RAI; (b) the tumor tissue has lost the ability to concentrate RAI after previous evidence of RAI avid disease; (c) RAI is concentrated in some lesions but not in others; (d) disease progresses despite significant concentration of RAI; (e) no remission with an accumulated dose of radioactive iodine over 600 mCi; (f) lack of ability of the tumor to concentrate sufficient RAI for tumoricidal effect, based on lesional dosimetry*.

4 *NOS: Newcastle-Ottawa Scale*.

5 *NA: not available*.

6 *PTC: papillary thyroid carcinoma*.

7 *FTC: follicular thyroid carcinoma*.

8 *PDTC: poorly differentiated thyroid carcinoma*.

9 *Median (interquartile range)*.

**Figure 2:**
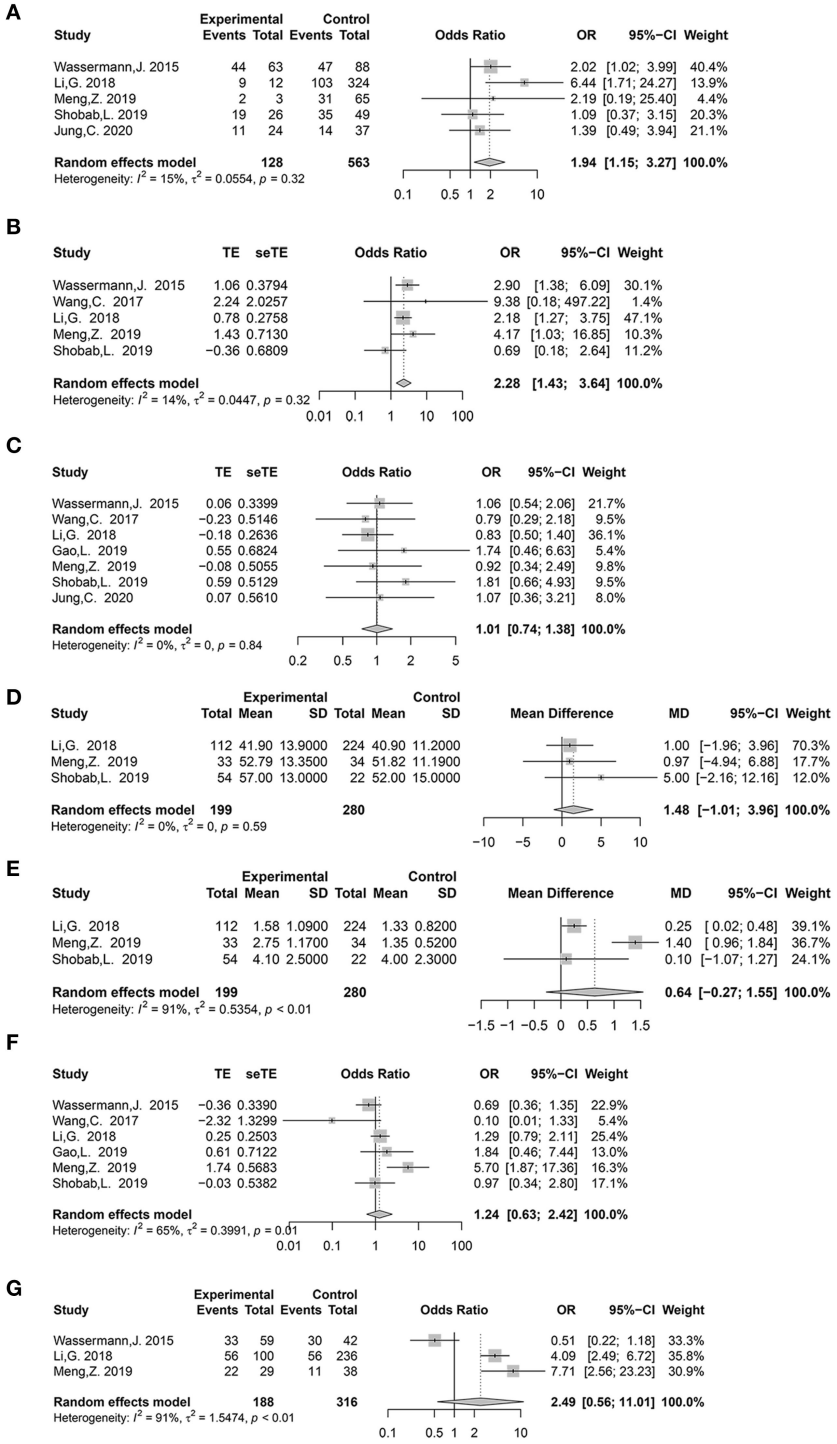
Forest plot detailing odds ratio and mean difference with 95% confidence intervals (CI) for the effect of histological subtype **(A)**, extrathyroidal extension **(B)**, gender **(C)**, age (years) **(D)**, tumor size (cm) **(E)**, multifocality **(F)**, and lateral lymph node metastasis **(G)**.

**Figure 3:**
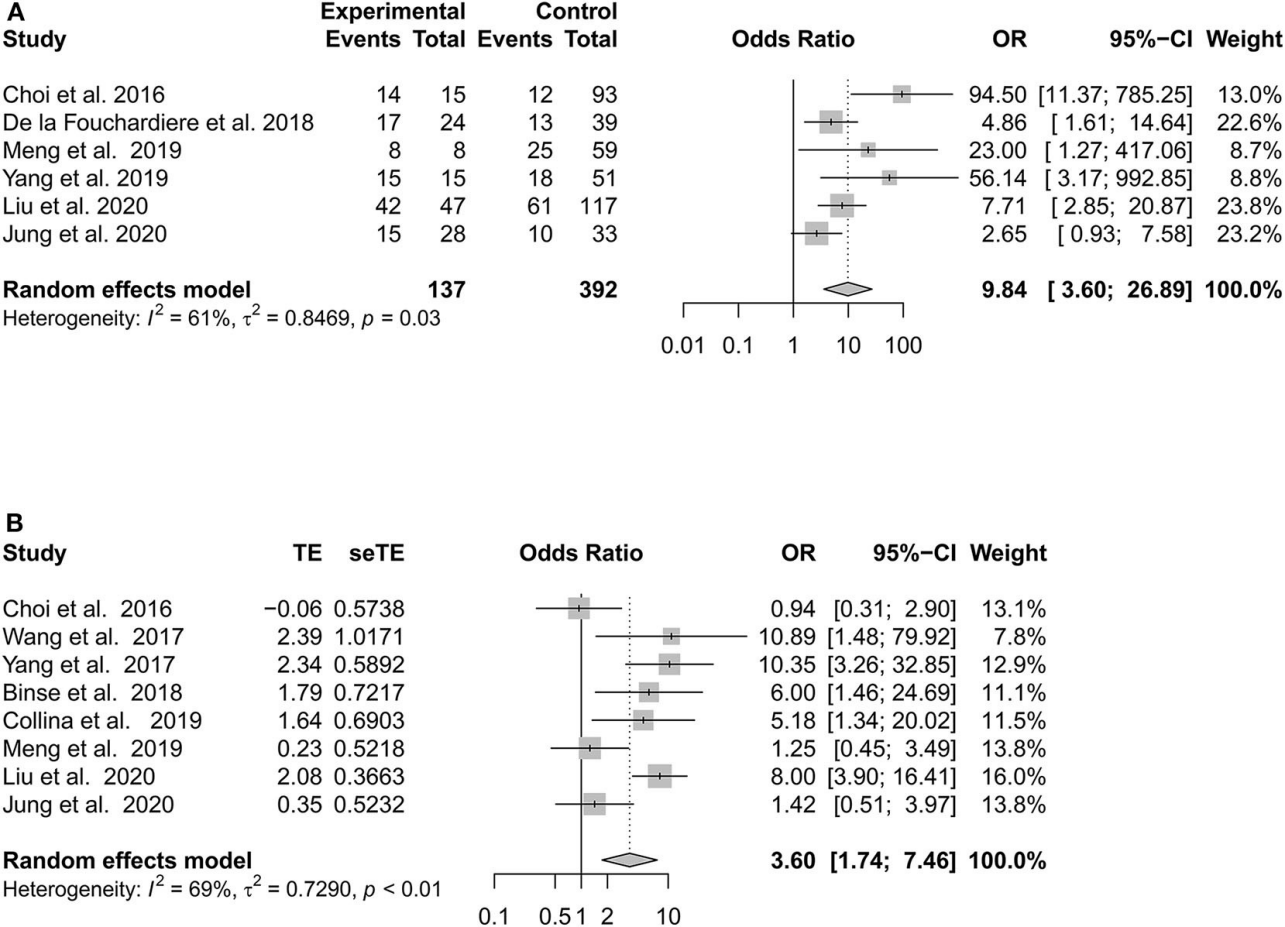
Forest plot detailing the odds ratios with 95% confidence intervals for the effect of *TERT* promoter mutation **(A)** and *BRAF*^*V*600*E*^ mutation **(B)**.

Nine potential risk factors were analyzed to pool the log OR or MD with a 95% CI: age, sex, histological subtype, tumor size, multifocality, extrathyroidal extension (ETE), lateral lymph node metastasis (LLNM), *TERT* promoter (*TERT*p) mutation, and *BRAF*^*V*600*E*^ mutation (Supplementary Tables 2, 3). In the meta-analysis, statistically significant clinical predictors were shown, as follows: histological subtype (*p* = 0.01, OR: 1.94, 95% CI: 1.15–3.27) (Figure 2A) and ETE (*p* < 0.01, OR: 2.28, 95% CI: 1.43–3.64) (Figure 2B). At the molecular level, significant effects were also found for both the TERTp mutation (*p* < 0.01, OR: 9.84, 95% CI: 3.60–26.89) and BRAFV600E mutation (*p* < 0.01, OR: 3.60, 95% CI: 1.74–7.46) (Figure 3). However, there was no statistical significance in sex (*p* = 0.95, OR: 1.01, 95% CI: 0.74–1.38) (Figure 2C), age (*p* = 0.24, MD: 1.48 years, 95% CI: −1.01 to 3.96) (Figure 2D), tumor size (*p* = 0.17, MD: 0.64 cm, 95% CI: −0.27 to 1.55) (Figure 2E), multifocality (*p* = 0.53, OR:1.24,95% CI: 0.63–2.42) (Figure 2F), or LLNM (*p* = 0.23, OR: 2.49, 95% CI: 0.56–11.01) (Figure 2G).

### Subgroup Analysis

Heterogeneities were obvious for the following five features: tumor size (*I*^2^ = 91%, *p* < 0.01), LLNM (*I*^2^ = 91%, *p* < 0.01), multifocality (*I*^2^ = 65%, *p* = 0.01), *BRAF*^*V*600*E*^ mutation (*I*^2^ = 69%, *p* < 0.01), and *TERT*p mutation (*I*^2^ = 61%, *p* = 0.03) (Supplementary Tables 2, 3). Due to the limited number of studies included, we performed subgroup analyses for the latter three to explore whether heterogeneity might be caused by countries and recruitment periods (Supplementary Table 4 and Supplementary Figure 1).

#### Multifocality of Different Countries

**Figure 4:**
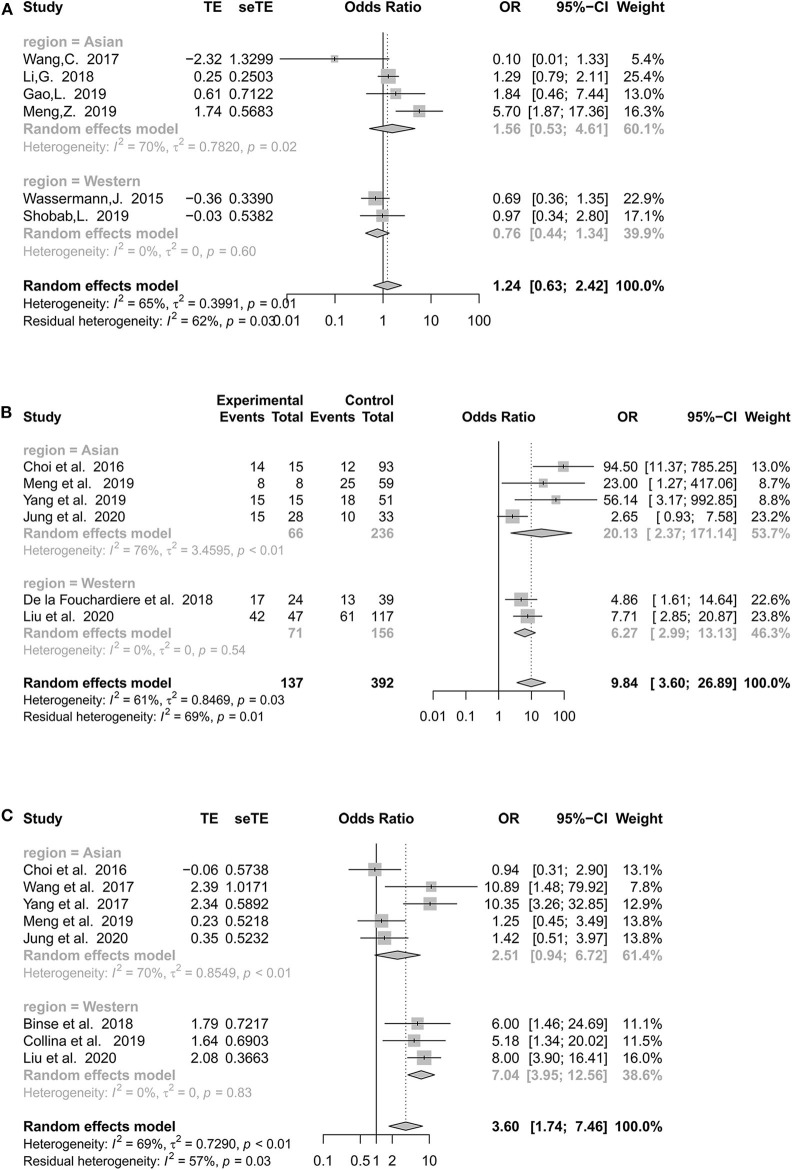
Forest plot detailing the odds ratios with 95% confidence intervals for the effect of multifocality **(A)**, *TERT* promoter mutation **(B)**, and *BRAF*^*V*600*E*^ mutation **(C)** according to different regions.

When subanalyzing multifocality, Asian studies continued to demonstrate moderate heterogeneity (*I*^2^ = 70%, *p* = 0.02), whereas heterogeneity showed a marked decrease in Western countries (*I*^2^ = 0%, *p* = 0.60) (Figure 4A). Both Asian (*p* = 0.43) and Western countries (*p* = 0.35) demonstrated a statistically insignificant association between multifocality and RR-DTC.

#### *TERT*p Mutation of Different Countries

National classification did not decrease the heterogeneity of *TERT*p mutations in the Asian group (*I*^2^ = 76%, *p* < 0.01), and the pooled estimates remained positive (OR: 20.13, 95% CI: 2.37–171.14, *p* < 0.01). Interestingly, there was no significant heterogeneity in Western countries (Figure 4B).

#### *BRAF* Mutations in Different Countries

Asian populations continued to demonstrate considerable heterogeneity, with an *I*^2^ of 70%. Asian studies (OR: 2.51, 95% CI: 0.94–6.72; *p* = 0.07) failed to show a significant association between the *BRAF*^*V*600*E*^ mutation and RR-DTC (Figure 4C). However, the heterogeneity of Western studies was 0% (OR: 7.04, 95% CI: 3.95–12.56, *p* < 0.01).

#### Genetic Factors of Different Recruitment Periods of Study

As shown in Table 1, the recruitment period differs in each study. We divided the data into two parts, before and after 2011, based on the median year of the inclusion time of each study. It showed that there was no obvious improvement in the heterogeneities of both *TERT*p and *BRAF*^*V*600*E*^ mutations (Supplementary Figure 1).

### Cumulative Meta-Analysis of Genetic Factors

**Figure 5:**
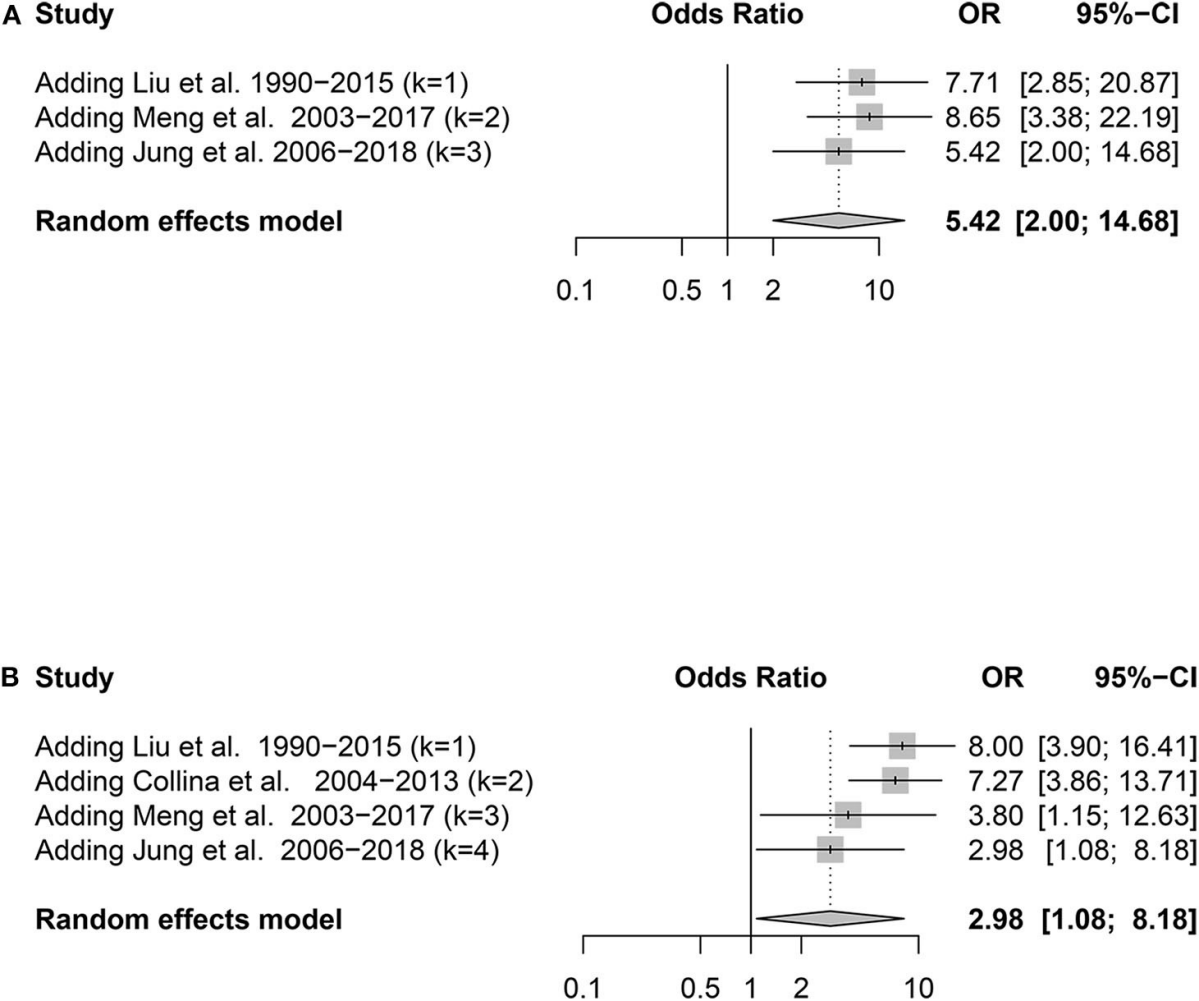
Forest plot detailing the odds ratios with 95% confidence intervals for the effect of *TERT* promoter mutation **(A)** and *BRAF*^*V*600*E*^ mutation **(B)** in the cumulative meta-analysis.

To determine whether the predictive ability of *TERT*p and *BRAF*^*V*600*E*^ mutation changes over time, we performed a cumulative meta-analysis depending on the main recruitment periods. The OR of the *BRAF*^*V*600*E*^ mutation obviously declined over time, while there was no clear tendency in the *TERT*p mutation (Figure 5).

### Sensitivity Analysis

We performed a leave-one-out sensitivity analysis to evaluate the robustness of pooled ORs and MDs for all nine factors (Supplementary Figure 2). The effect size of age, sex, histological subtype, ETE, and *BRAF*^*V*600*E*^ mutation revealed no significant change, indicating the stability of these risk factors.

The heterogeneity of tumor size was high (*I*^2^ = 91%), but there was no heterogeneity after removing the study (*I*^2^ = 0%) (11). The pooled OR was recalculated, and it was a statistically significant result (before: MD 0.64 cm, 95% CI: −0.27 to 1.55, *p* = 0.17; after MD 0.24 cm, 95% CI: 0.02–0.47, *p* = 0.03). For multifocality, we found that one study (11) could be the reason for the high heterogeneity, because the result became stable after excluding it (before: *I*^2^ = 65%; after: *I*^2^ = 34%). However, the *p*-value was still statistically insignificant despite the fact that a fixed-effect model was used to replace the former random-effects model, which indicated that multifocality was not a proper predictor of the occurrence of RR-DTC. During the sensitivity analysis of LLNM, it was found that the deletion of one study (18) turned the high heterogeneity (*I*^2^ = 91%) into no heterogeneity (*I*^2^ = 5%) with a statistically significant outcome (before: OR 2.49, 95% CI: 0.56–11.01, *p* = 0.23; after: OR 4.61, 95% CI: 2.84–7.48, *p* < 0.01).

At the molecular level, for *TERT*p mutation, when we removed one study (9), the pooled log OR of RR-DTC decreased from 9.84 (95% CI: 3.60–26.89, *p* < 0.01) to 6.13 (95% CI: 2.78–13.47, *p* < 0.01), and the statistical heterogeneity declined (before: *I*^2^ = 61%; after: *I*^2^ = 35%), which suggested that it could be the source of heterogeneity. However, there were few changes in the statistical heterogeneity after removing the literature sequentially in the sensitivity analysis of the *BRAF*^*V*600*E*^ mutation, indicating its robust outcome.

## Discussion

Recently, RR-DTC has become a tricky problem as a result of the increasing number of patients with PTC around the world. The present study of 11 observational studies synthesized and explored potential risk factors for RR-DTC. We found four risk factors, as follows: histological subtype, ETE, *TERT*p mutation, and *BRAF*^*V*600*E*^ mutation.

Generally, grades of malignancy were related to age and sex in DTCs, wherein younger and female patients have a better prognosis. Some have reported that RAI avidity has a significant inverse correlation with age (12, 13, 20, 21). Males with RR-DTC tend to have poorer survival than females (4). However, our results showed that demographics, including age and sex, were not statistically significant. A possible explanation is that most of the literature included was age- and sex-matched. Nevertheless, to our experience, young age may be a protective factor, but further investigations of the association between demographics and RR-DTC are required. In addition, publication bias could not be ignored when making conclusions.

Histology has been known as an important predictor of prognosis in PTC. In patients with RR-DTC, some studies illustrated that FTC, Hürthle cell, and poorly differentiated thyroid carcinoma (PDTC) had a higher possibility of being refractory to radioiodine treatment (22–25). It was also found that there was a considerable amount of histologic plasticity between the primary lesions and metastases, indicating that tumor cells became less differentiated during the progression of ^131^I treatment (26). In the present study, classic and follicular variant PTCs were less aggressive histological subtypes, which were classified as the low-risk histological group, while the others were categorized as the high-risk histological group: tall cell variant PTC, sclerosing diffuse PTC, hobnail variant PTC, FTC (including Hürthle cell), and PDTC. The meta-analysis further confirmed that histological subtype was a predictor of RR-DTC (OR: 1.94, 95% CI: 1.15–3.27, *p* = 0.01) with low heterogeneity (*I*^2^ = 15%, *p* = 0.32). Sensitivity analysis also assessed the stability of the association.

Generally, a large tumor size is a useful feature for indicating the worse prognosis of PTC (27, 28), but it remains unknown in RR-DTC. Here, we drew the opposite conclusion. Three studies including 100 RR-DTCs and 115 controls were analyzed in this part: two from China (11, 17) and one from America (12). In the sensitivity analysis, deletion of one study (11) changed the high heterogeneity into no heterogeneity. Nevertheless, regardless of whether sensitivity analysis was performed, the MD between patients with RR-DTC and controls was minor (before sensitivity analysis: 0.64 cm; after sensitivity analysis: 0.24 cm). Thus, it has relatively low clinical significance and value. Of course, it is necessary to investigate and further prove this idea.

There is no doubt that ETE correlates with poor survival of PTCs (29–31), whereas the value of ETE in RR-DTC is unclear. The present study illustrated that ETE had a positive relationship with the development of RR-DTC (*p* < 0.01, OR: 2.28, 95% CI: 1.43–3.64), and the sensitivity analysis confirmed the robustness of the results. However, ETE is associated with other factors, such as histological subtype, the location of the primary lesion, and the timing of diagnosis. For example, the ETE probability of tumors is higher in the isthmus than in the thyroid gland lobe due to its anatomical position, while the incidence of RR-DTC might be similar on the assumed premise of the same clinical stage and the same histology. Thus, ETE could be a risk factor, but it needs to be combined with other factors when making clinical decisions and strategies, and further studies are urgently needed.

Both multifocality and LLNM had no statistical association with the occurrence of radioiodine resistance in the overall study. However, further analysis suggested a positive relationship between LLNM and RR-DTC (OR 4.61, 95% CI: 2.84–7.48, *p* < 0.01, *I*^2^ = 5%) by removing one study (18). This might be explained by the fact that lymph nodes were not evaluated by a pathologist (Nx) in more than one third of patients in both the refractory DTC and the control groups in one study (18). Therefore, the great difference would be reconciled if this part of the data were complete. Although the subgroup analysis for LLNM was not conducted due to the similar study number limitation as tumor size, the outcome was relatively reliable because the literature remaining (11, 17) contained many patients, specifically 403 patients with DTC, of whom 145 were diagnosed with RR-DTC. LLNM is a predictor of the prognosis of DTC (32–34), which reflects the aggressiveness of tumors similar to ETE. Unfortunately, there are few studies relevant to LLNM and RR-DTC at the moment, and only a tendency was shown here. More studies related to metastatic lymph nodes are required to prove this concept.

Molecular analysis provides useful insight into the role of predicting the occurrence of RR-DTC, and *BRAF*^*V*600*E*^/*TERT*p mutations are associated with greater aggressiveness of TC. Liu et al. (35) reported that the *TERT*p mutation was a malignant molecular marker for follicular cell-derived thyroid carcinoma. Possible mechanisms behind the association between genetic mutations and RR-DTC have been found. First, there are frequent mutations in C228T and C250T, two prevalent hot positions, which may induce hyperactivity of *TERT* and lead to over proliferation and carcinogenesis (36, 37). Second, one hallmark of dedifferentiation of DTC is impairment of NIS function (38). Tavares reported a genetic background in which *BRAF*^*V*600*E*^, *TERT*p, and *NRAS* inversely correlated with NIS expression (39). Currently, the *BRAF*^*V*600*E*^ mutation has been found to repress NIS expression in two ways: the TGFβ-Smad3–PAX8 pathway and histone deacetylation of NIS (40–42). The meta-analysis indicated that positive *BRAF*^*V*600*E*^ (*p* < 0.01, OR: 3.60, 95% CI: 1.74–7.46) and *TERT*p (*p* < 0.01, OR: 9.84, 95% CI: 3.60–26.89) mutations were risk factors for RR-DTC. However, according to the results of the cumulative meta-analysis, we found an obvious decreasing tendency of the prediction effect of the *BRAF*^*V*600*E*^ mutation, which could be used to explain the considerable heterogeneity of the *BRAF*^*V*600*E*^ mutation. Over the past few decades, an increased incidence of *BRAF*^*V*600*E*^ mutations has been reported in PTCs, especially classic papillary PTCs (43–45). In the meantime, the incidence of RR-DTC remains stable relatively. Thus, the RAIR predictive ability of *BRAF*^*V*600*E*^ mutations may decline because of the attenuation effect conducted by the increasing amount of PTCs with *BRAF*^*V*600*E*^ mutations. The possible reason is the great advances in genetic testing technology with higher sensitivity and specificity currently so that we could detect the *BRAF*^*V*600*E*^ mutations we could not before. Additionally, the predictive role of *BRAF* should be further considered in patients with papillary thyroid microcarcinoma (PTMC), defined as a tumor of 1 cm or less in size. Based on the available information, the 2015 ATA suggested that *BRAF* had a limited role in increasing the recurrence risk of patients with PTMC (46). Unfortunately, we lacked PTMC data to perform a subgroup analysis of PTMC in our study. A possible reason why there were no data could be the good prognosis and the quite low recurrence rate of PTMC, 2–6% locoregional recurrence, and 1–2% distant recurrence (47, 48). Thus, it is too rare for a patient with PTMC to develop RR-DTC. Hence, the *BRAF*^*V*600*E*^ would be a predictor of RR-DTC for patients with PTC except PTMC. Interestingly, according to our subgroup and sensitivity analyses of *TERT*p mutation, a Korean study (9) was found to increase the heterogeneity and the pooled OR considerably (before: OR 9.84, *p* < 0.01, 95% CI: 3.60–26.89, *I*^2^ = 61%; after: OR 6.13, *p* < 0.01, 95% CI: 2.78–13.47, *I*^2^ = 35%). The possible explanation could be that the control group was the RAIA group without distant metastasis, within whom the *TERT*p mutation would be much lower. It could be improved if the control group had a similar ratio of local recurrence and distant metastasis to the RAIR group.

Moreover, when further considering the coexistence of *TERT*/*BRAF* mutations, the predictive power seems to be better. Vuong reported that concurrent *TERT*/*BRAF* mutations were associated with increased tumor aggressiveness than were PTCs with *BRAF*^*V*600*E*^ or *TERT*p mutation alone, and *TERT*p mutation was more high risk than *BRAF*^*V*600*E*^ in terms of tumor aggressiveness (49). It was also reported that distant metastases showed enrichment in *TERT*p mutations and a decrease in *BRAF* mutations in comparison with paired primary tumors (50, 51). In addition, Yang et al. (8) indicated a greater proportion of *TERT*p mutations in the RAIR group than in the *BRAF* group. Nevertheless, it remains unclear whether *TERT*p mutation dominates prognosis prediction or whether there are any other better predictive genetic factors, such as RAS. In summary, both the *BRAF*^*V*600*E*^ mutation and *TERT*p mutation were meaningful predictors for RR-DTC, and a joint evaluation was required. Once the coexistence of *TERT*/*BRAF* mutations is confirmed, high attention should be paid to RAIR patients.

Despite our efforts, several potential limitations should be noted in the meta-analysis. First, the small number of eligible studies restricted the analytical process. The majority of studies included were retrospective. Second, the definition and classifications of RR-DTC varied slightly between the included studies. However, the current classifications that we could find were not necessarily sufficient evidence for diagnosing radioiodine refractory disease, although they were clinically useful (52). Third, consideration of confounding factors varied across studies, and certain valuable factors were not consistently reported, such as race, TNM stage, number of RAI treatments, and cumulative dose of RAI. Hence, the subgroup analysis was limited, and we cannot restore heterogeneous sources more authentically and meticulously. Finally, our meta-analysis was based on study-level data but not individual participant data. Individual participant-level meta-analysis could provide more reliable risk estimates than the study-level meta-analysis.

## Conclusion

This meta-analysis indicated that high-risk histological subtypes, ETE, *TERT*p mutation, and *BRAF*^*V*600*E*^ mutation are related to the occurrence of iodine resistance in PTCs. High-risk histological subtypes include tall cell variant PTC, sclerosing diffuse PTC, hobnail variant PTC, FTC (including Hürthle cell), and PDTC. In contrast, tumor diameter and multifocality were not predictors according to our results. Meanwhile, other factors, including age, sex, and LLNM, may be useful and valuable predictors, but their values in RR-DTC need to be further investigated.

## Data Availability Statement

All datasets generated for this study are included in the article/Supplementary Material.

## Author Contributions

YW: conceptualization, supervision, and project administration. YL: methodology. YL, HJ, WX, XW, and BM: acquisition, analysis, curation, and validation of data. YL, HJ, WX, XW, BM, and TL: writing—original draft preparation, review and editing. All authors contributed to the article and approved the submitted version.

## Conflict of Interest

The authors declare that the research was conducted in the absence of any commercial or financial relationships that could be construed as a potential conflict of interest.

## Supplementary Material

Supplementary Figure 1 Forest plot detailing *TERT* promoter mutation **(A)** and *BRAF*^*V*600*E*^ mutation **(B)** according to different recruitment periods (before 2011 and after 2011).

Supplementary Figure 2 Forest plot detailing the leave-one-out sensitivity analysis for the effect of sex **(A)**, age (years) **(B)**, tumor size (cm) **(C)**, multifocality **(D)**, histological subtype **(E)**, extrathyroidal extension **(F)**, lateral lymph node metastasis **(G)**, *BRAF*^*V*600*E*^ mutation **(H)**, and *TERT* promoter mutation **(I)**.

Supplementary Table 1 Quality of eligible studies (Newcastle-Ottawa Scale).

Supplementary Table 2 Analysis of the categorical variables for patients with RR-DTC.

Supplementary Table 3 Analysis of the continuous variables for patients with RR-DTC.

Supplementary Table 4 Analysis of factors of RR-DTC patients in different regions.
