# Supplementary figures and images for: Clinical, Pathological, and Molecular Characteristics Correlating to the Occurrence of Radioiodine Refractory Differentiated Thyroid Carcinoma: A Systematic Review and Meta-Analysis
Source: Front Oncol. 2020 Sep 30;10:549882. doi: 10.3389/fonc.2020.549882 (PMC7561400; doi:10.3389/fonc.2020.549882)

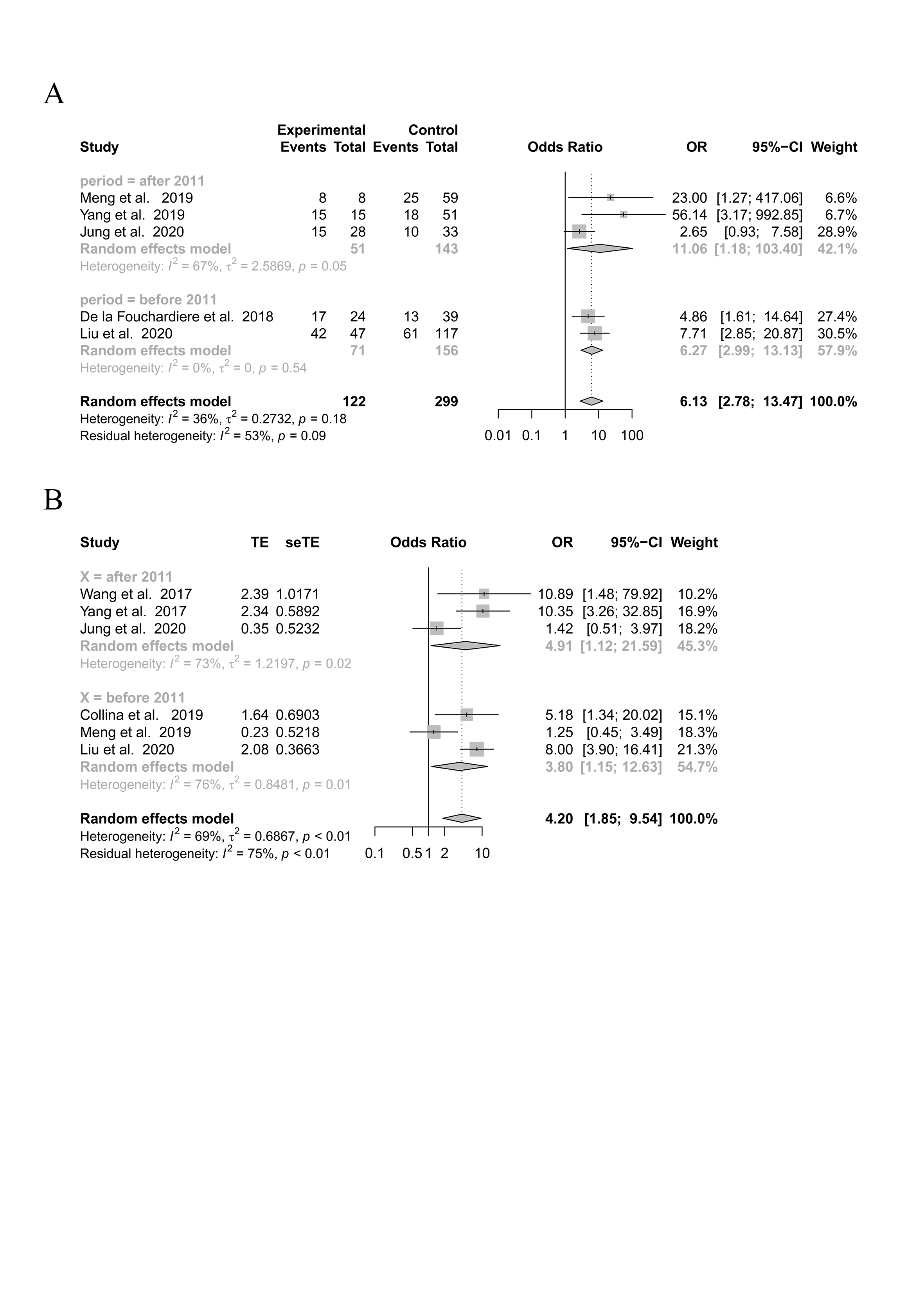

Supplement: Supplementary Figure 1 — Forest plot detailing TERT promoter mutation (A) and BRAFV600E mutation (B) according to different recruitment periods (before 2011 and after 2011). [file Image_1.TIF]

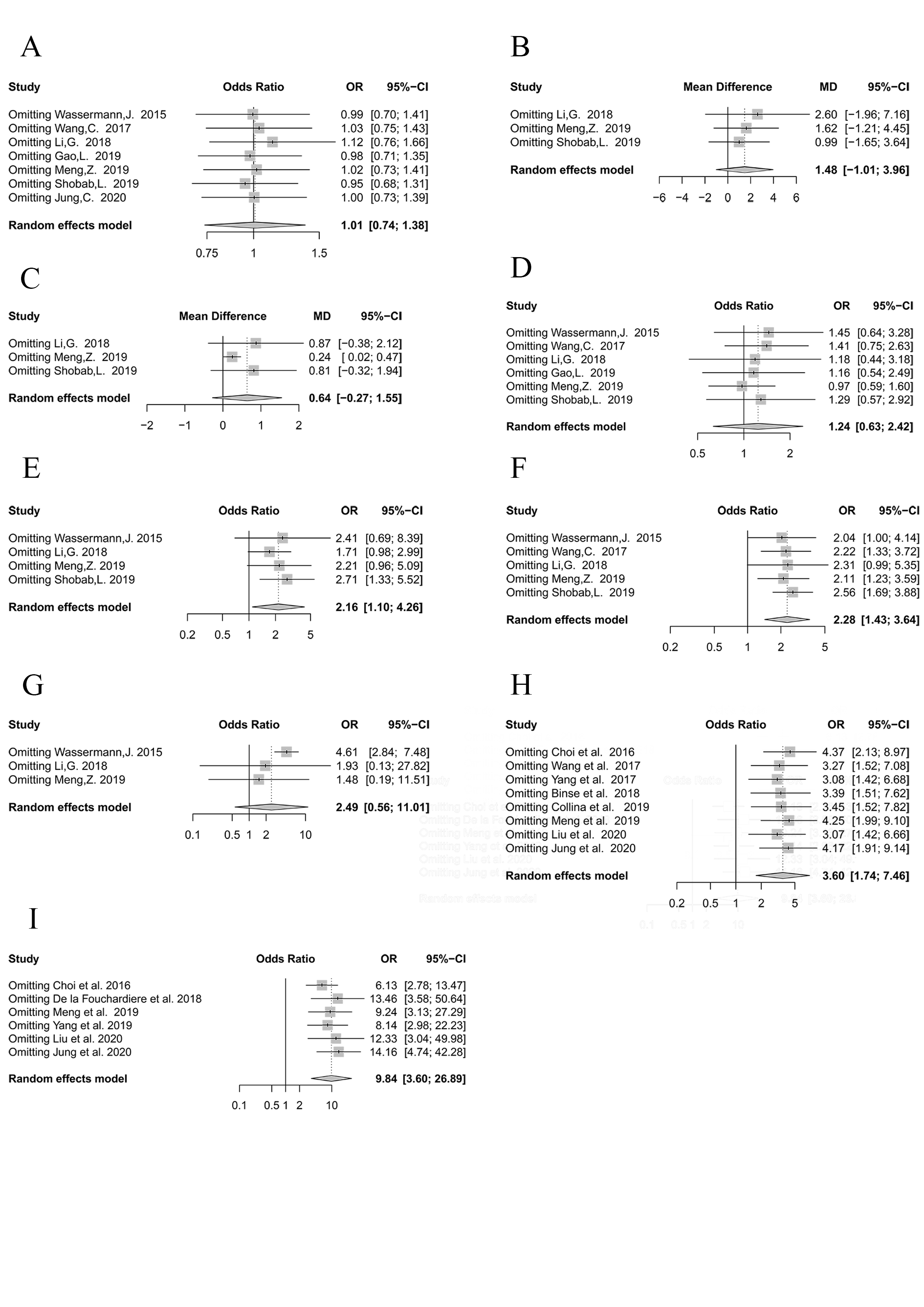

Supplement: Supplementary Figure 2 — Forest plot detailing the leave-one-out sensitivity analysis for the effect of sex (A), age (years) (B), tumor size (cm) (C), multifocality (D), histological subtype (E), extrathyroidal extension (F), lateral lymph node metastasis (G), BRAFV600E mutation (H), and TERT promoter mutation (I). [file Image_2.TIF]
