# Supplementary material for: Clinical, Pathological, and Molecular Characteristics Correlating to the Occurrence of Radioiodine Refractory Differentiated Thyroid Carcinoma: A Systematic Review and Meta-Analysis
Source: Front Oncol. 2020 Sep 30;10:549882. doi: 10.3389/fonc.2020.549882 (PMC7561400; doi:10.3389/fonc.2020.549882)
Supplement: Supplementary Table 1 — Quality of eligible studies (Newcastle-Ottawa Scale). [file Table_1.docx]

Supplementary Tables 1–4

**Table S1.**  Quality of eligible studies (Newcastle-Ottawa Scale)

| **Study** | **Selection** | | | | **Compara-bility** | **Exposure** | | | **Scores** |
| --- | --- | --- | --- | --- | --- | --- | --- | --- | --- |
|  | **Case**  **definition** | **Representa-**  **tiveness** | **Control**  **Selection** | **Control**  **Definition** |  | **Ascertainment**  **of Exposure** | **Same Method of**  **Ascertainment**  **for Cases and Controls** | **Non-**  **Response**  **Rate** |  |
| Binse,I.2018 | 1 | 1 | 0 | 1 | 2 | 1 | 1 | 0 | 7 |
| Yang,X.2017 | 1 | 1 | 0 | 1 | 2 | 1 | 1 | 0 | 7 |
| Choi Sun,E.2016 | 1 | 1 | 0 | 1 | 1 | 1 | 1 | 0 | 6 |
| Gao,L 2019 | 1 | 1 | 0 | 1 | 2 | 1 | 1 | 1 | 8 |
| Meng,Z. 2019 | 1 | 1 | 0 | 1 | 2 | 1 | 1 | 0 | 7 |
| Shobab,L. 2019 | 1 | 1 | 0 | 1 | 2 | 1 | 1 | 0 | 7 |
| Wang,C. 2017 | 1 | 1 | 0 | 1 | 2 | 1 | 1 | 0 | 7 |
| Collina,F. 2019 | 1 | 1 | 0 | 1 | 2 | 1 | 1 | 0 | 7 |
| De la Fouchardiere,C. 2018 | 1 | 1 | 0 | 1 | 2 | 1 | 1 | 0 | 7 |
| Li,G. 2018 | 1 | 1 | 0 | 1 | 2 | 1 | 1 | 0 | 7 |
| Wassermann,J.2015 | 1 | 1 | 0 | 1 | 2 | 1 | 1 | 0 | 7 |
| Liu,J.2020 | 1 | 1 | 0 | 1 | 2 | 1 | 1 | 0 | 7 |
| Jung,C. 2020 | 1 | 1 | 0 | 1 | 2 | 1 | 1 | 0 | 7 |

**Table S2.** Analysis of the categorical variables for patients with RR-DTC.

| **Factors** | **No. of studies** | **Results** | | | **Model** | **Heterogeneity** | |
| --- | --- | --- | --- | --- | --- | --- | --- |
|  |  | **OR**^3^ | **95% CI**^4^ | **p value** |  | **p value** | **I^2^ (%)** |
| Gender | 7 | 1.01 | 0.74-1.38 | 0.95 | Random | 0.84 | 0 |
| Histological subtype | 5 | 1.94 | 1.15-3.27 | 0.01 | Random | 0.32 | 15 |
| Multifocality | 6 | 1.24 | 0.63-2.42 | 0.53 | Random | 0.01 | 65 |
| ETE^1^ | 5 | 2.28 | 1.43-3.64 | <0.01 | Random | 0.32 | 14 |
| LLNM^2^ | 3 | 2.49 | 0.56-11.01 | 0.23 | Random | <0.01 | 91 |
| *BRAF^V600E^* mutation | 8 | 3.6 | 1.74-7.46 | <0.01 | Random | <0.01 | 69 |
| *TERT* promoter mutation | 6 | 9.84 | 3.60-26.89 | <0.01 | Random | 0.03 | 61 |

^1^ ETE: extrathyroidal extension.

^2^ LLNM: lateral lymph node metastasis.

^3^ OR: odds ratio.

^4^95% CI: 95% confidence interval.

**Table S3.** Analysis of the continuous variables for patients with RR-DTC.

| **Factors** | **No. of studies** | **Results** | | | **Model** | **Heterogeneity** | |
| --- | --- | --- | --- | --- | --- | --- | --- |
|  |  | **MD**^1^ | **95% CI^2^** | **p value** |  | **p value** | **I^2^ (%)** |
| Age (years) | 3 | 1.48 | -4.97 | 0.24 | Random | 0.59 | 0 |
| Tumor size (cm) | 3 | 0.64 | -1.82 | 0.17 | Random | <0.01 | 91 |

^1^ MD: mean difference.

^2^ 95% CI: 95% confidence interval.

**Table S4.** Analysis of factors of RR-DTC patients in different regions

| **Factors** | **No. of studies** | **Cases** | **Result** | | **Heterogeneity** | |
| --- | --- | --- | --- | --- | --- | --- |
|  |  |  | **OR (95% CI)^1^** | **p Value** | **I^2^ (%)** | **p Value** |
| Multifocality |  |  |  |  |  |  |
| Asian | 4 | 590 | 1.56 (0.53,4.61) | 0.43 | 70 | 0.02 |
| Western | 2 | 229 | 0.76 (0.44,1.34) | 0.35 | 0 | 0.6 |
| *TERT* promoter mutation | |  |  |  |  |  |
| Asian | 4 | 302 | 20.13 (2.37,171.14) | <0.01 | 76 | <0.01 |
| Western | 2 | 227 | 6.27 (2.99,13.13) | <0.01 | 0 | 0.54 |
| *BRAF^V600E^* mutation |  |  |  |  |  |  |
| Asian | 5 | 417 | 2.51 (0.94,6.72) | 0.07 | 70 | <0.01 |
| Western | 3 | 317 | 7.04 (3.95,12.56) | <0.01 | 0 | 0.83 |

^1^ OR (95% CI): odds ratio (95% confidence interval).
